# Associations of Serum Uromodulin and Its Genetic Variants With Blood Pressure and Hypertension in Chinese Adults

**DOI:** 10.3389/fcvm.2021.710023

**Published:** 2021-11-17

**Authors:** Yang Wang, Ming-Fei Du, Shi Yao, Ting Zou, Xiao-Yu Zhang, Gui-Lin Hu, Chao Chu, Yue-Yuan Liao, Chen Chen, Dan Wang, Qiong Ma, Ke-Ke Wang, Yue Sun, Ze-Jiaxin Niu, Rui-Chen Yan, Yu Yan, Hao-Wei Zhou, Hao Jia, Wei-Hua Gao, Hao Li, Chun-Hua Li, Fang-Yao Chen, Ke Gao, Jie Zhang, Robert Safirstein, Feng Wang, Tie-Lin Yang, Jian-Jun Mu

**Affiliations:** ^1^Department of Cardiovascular Medicine, First Affiliated Hospital of Xi'an Jiaotong University, Xi'an, China; ^2^Key Laboratory of Molecular Cardiology of Shaanxi Province, Xi'an, China; ^3^Key Laboratory of Biomedical Information Engineering of Ministry of Education, Biomedical Informatics & Genomics Center, School of Life Science and Technology, Xi'an Jiaotong University, Xi'an, China; ^4^Department of Cardiology, Northwest Women's and Children's Hospital of Xi'an Jiaotong University Health Science Center, Xi'an, China; ^5^Department of Cardiology, Xi'an No.1 Hospital, Xi'an, China; ^6^Department of Critical Care Medicine, First Affiliated Hospital of Xi'an Jiaotong University, Xi'an, China; ^7^Department of Ophthalmology, Xi'an People's Hospital, Xi'an, China; ^8^Department of Epidemiology and Biostatistics, School of Public Health, Xi'an Jiaotong University Health Science Center, Xi'an, China; ^9^Department of Cardiology, Xi'an People's Hospital, Xi'an, China; ^10^Department of Medicine, Yale University School of Medicine, New Haven, CT, United States; ^11^Department of Nephrology, Jiangsu University Affiliated Shanghai Eighth People's Hospital, Shanghai, China

**Keywords:** gene polymorphism, hypertension, uromodulin (*UMOD*), longitudinal change, blood pressure

## Abstract

**Background:** Uromodulin, also named Tamm Horsfall protein, has been associated with renal function and regulation of sodium homeostasis. We aimed to examine the associations of serum uromodulin levels and its genetic variants with longitudinal blood pressure (BP) changes and hypertension incidence/risk.

**Methods:** A total of 514 participants from the original Baoji Salt-Sensitive Study cohort were genotyped to examine the associations of genetic variations in uromodulin gene with the longitudinal BP changes and the incidence of hypertension over 8 years of follow-up. In addition, 2,210 subjects from the cohort of Hanzhong Adolescent Hypertension Study were used to investigate the relationships between serum uromodulin levels and the risk of hypertension.

**Results:** SNPs rs12917707 and rs12708631 in the uromodulin gene were significantly associated with the longitudinal BP changes over 8 years of follow-up. SNP rs12708631 was significantly associated with the incidence of hypertension over 8 years. In addition, gene-based analyses supported the associations of uromodulin gene with the longitudinal BP changes and hypertension incidence in Baoji Salt-Sensitive Study cohort. Furthermore, serum uromodulin levels in the hypertensive subjects were lower than in the normotensive subjects (25.5 ± 1.1 *vs*. 34.7 ± 0.7 ng/mL). Serum uromodulin levels decreased gradually as BP levels increased (34.6, 33.2, 27.8, and 25.0 ng/mL for subjects with normotension, high-normal, grade 1 hypertension, and grade 2 hypertension, respectively). Serum uromodulin was significantly associated with the lower risk of hypertension [0.978 (0.972–0.984)] in Hanzhong Adolescent Hypertension Study cohort.

**Conclusion:** This study shows that uromodulin is associated with blood pressure progression and development of hypertension.

## Introduction

Hypertension, due to its high prevalence and its associated risk of cardiovascular disease and all-cause mortality, is considered as a major worldwide public health challenge (1, 2). Even a slight increase in blood pressure (BP) increases the risk of cardiovascular events (3). Sodium (Na^+^) homeostasis plays an important role in the regulation of BP, and small changes in the rate of its reabsorption may cause significant changes in Na^+^ excretion, leading to disturbances in the Na^+^ balance and extracellular fluid volume and ultimately to hypertension (4, 5).

Uromodulin, also known as Tamm-Horsfall protein, is a 95 kDa glycoprotein. Encoded by the *UMOD* gene located on chromosome 16p12.3 (6, 7), uromodulin is synthesized mainly by the thick ascending limb (TAL) and early distal convoluted tubule in the kidney (8). A large amount of uromodulin is secreted into the urinary tract and exerts anti-inflammatory, anti-infective and electrolyte treatment effects (9–12). In addition to its apical secretion, a small proportion of uromodulin is secreted from the basolateral side into the interstitial space and enters the blood (13). The physiological function of blood uromodulin is unknown, but it has emerged as a potential biomarker for renal function (14, 15). In addition, prior studies showed that genetic variants in the *UMOD* gene were associated with BP phenotypes and hypertension (16–18). However, no study has yet explored the associations of common variants in the *UMOD* gene with the longitudinal BP changes or the incidence of hypertension. Furthermore, data are scarce about the relationship between serum uromodulin levels and the risk of hypertension.

In the present study we used single marker–and gene–based analyses to prospectively evaluate the associations of *UMOD* gene with the longitudinal BP changes and the incidence of hypertension in a family-based cohort. In addition, we also used our large cross-sectional cohort to explore the possible relationships between serum uromodulin levels and the risk of hypertension.

## Methods

The entire study consisted of two parts: (1) a longitudinal cohort study to explore the associations of *UMOD* gene with longitudinal BP changes and hypertension incidence; and (2) a cross-sectional cohort study to examine the relationships between serum uromodulin levels and the risk of hypertension.

### Protocol 1: A Longitudinal Cohort Study to Explore the Associations of *UMOD* Gene With the Longitudinal BP Changes and the Incidence of Hypertension

Assembled from April to November 2004, the Baoji Salt-Sensitive Study cohort consists of 514 adults from 124 families in seven villages in Baoji, Shaanxi, China. The detailed design of this study has been published previously (19–24). To explore the associations of *UMOD* genetic variations with longitudinal BP changes and hypertension incidence, we followed up this cohort in 2009 and 2012. During each follow-up visit, a 3-day examination was performed as in 2004. Three BP measurements were obtained during each 3-day follow-up visit, and the mean of the nine BP measurements was used for the current analysis.

This protocol was approved by the Ethics Committee of the First Affiliated Hospital of Xi'an Jiaotong University, and adhered to the principles of the Declaration of Helsinki. Written informed consents were obtained from each participant (Trial registration number: NCT02915315. ClinicalTrials.gov).

### Protocol 2: A Cross-Sectional Cohort Study to Examine the Relationships Between Serum Uromodulin Levels and the Incidence of Hypertension

In 1987, we established the cohort of Hanzhong Adolescent Hypertension Study, which was an ongoing prospective, population-based cohort study of 4,623 adolescents aimed to screen for cardiovascular risk factors that originate in childhood and adolescence. Detailed information of study has been published elsewhere (25–30). To explore the association of serum uromodulin levels with the risk of hypertension, we used cross-sectional analysis of the latest follow-up data in 2017. The participant selection process is described in Supplementary Figure 1. 2,780 participants were examined in 2017. After excluding 566 participants with missing data (e.g., serum or urinary biochemistry) and four participants with self-reported history of coronary heart disease, renal failure or stroke, 2,210 participants were included for the final analysis.

The study protocol was reviewed and approved by the Ethics Committee of the First Affiliated Hospital of Xi'an Jiaotong University. This study followed the principles of the Declaration of Helsinki, and informed consent was obtained from study participants. The trial registration number was NCT02915315.

### BP Measurements

BP was measured in the sitting position by trained and certified observers using mercury sphygmomanometers. Smoking, drinking, coffee/tea and strenuous exercise were strictly prohibited for all participants at least 30 min before BP measurements. Systolic BP (SBP) and diastolic BP (DBP) were recorded at the first and fifth phases of the Korotkoff sounds. During the baseline survey and follow-ups, each subject needed to measure BP three times, and the average of nine BP measurements was calculated and recorded. The mean arterial pressure (MAP) was defined as DBP + [1/3 × (SBP – DBP)]. Pulse pressure (PP) was calculated as SBP – DBP. Hypertension was defined as SBP ≥ 140 mmHg, DBP ≥ 90 mmHg or use of antihypertensive drugs (31). We categorized all subjects into normotension, high-normal BP, grade 1 and grade 2 hypertension according to the 2020 International Society of Hypertension global hypertension practice guidelines (31). The subtypes of hypertension were further defined as isolated systolic hypertension (ISH), isolated diastolic hypertension (IDH), and systolic diastolic hypertension (SDH) in the absence of antihypertensive treatment (31).

### Blood Biochemical Analyses

Total cholesterol, high-density lipoprotein (HDL), low-density lipoprotein (LDL), triglycerides, alanine aminotransferase (ALT), aspartate aminotransferase (AST), serum creatinine, serum uric acid (SUA) and serum glucose levels were analyzed by an automatic biochemical analyzer (Hitachi, Tokyo, Japan) as described previously (23, 32–34). Serum uromodulin levels were measured by commercially available enzyme-linked immunosorbent assay (ELISA) kits (Cusabio Biotech, Wuhan, China). Five samples were randomly selected to evaluate intra and inter-assay coefficients of variation for uromodulin, which ranged from 2.3 to 5.9% and 3.7 to 6.8%, respectively.

### SNP Selection and Genotyping

Based on the Genome Variation Server database (http://gvs.gs.washington.edu/GVS147/) and the National Center for Biotechnology Information database (http://www.ncbi.nlm.nih.gov/projects/SNP), we selected 13 tagged SNPs in the *UMOD* gene (rs77875418, rs4293393, rs7193058, rs4997081, rs11859916, rs13333226, rs79245268, rs4632135, rs4383153, rs12708631, rs7198000, rs6497476 and rs12917707). Genomic DNA was isolated and purified from the whole blood samples by the GoldMag-Mini kits (GoldMag Co. Ltd. Xian, China). All the genotyping experiments were completed by CapitalBio (CapitalBio Corp, Beijing, China) as previously described (19–22, 24).

### Statistical Analyses

For the analyses in the longitudinal cohort study, we used PLINK software (version 1.9, http://zzz.bwh.harvard.edu/plink/) to test the quality control, including genotyping call rate, Mendelian consistency, minor allele frequency and Hardy-Weinberg equilibrium on parental SNP data. The associations of each SNP with BP phenotypes were examined in three genetic models (dominant, recessive and additive) by mixed-effect regression models using *lme* function in *nlme* R package. For the analyses of the incidence of hypertension, 51 participants with hypertension diagnosed at baseline were excluded. The additive associations of each SNP with hypertension incidence were examined by a generalized linear mixed model, which allows multi-level modeling when the response variable follows a binary distribution (e.g., incident hypertension). Baseline age, gender and body mass index (BMI) as fixed effects and familial correlation as a random effect were adjusted in the multivariable analysis using *glmer* function in *lme4* R package.

In addition, we used the truncated product method (TPM), which combines *P* values from each SNP association analysis, to evaluate the overall associations of *UMOD* gene with longitudinal BP changes and the incidence of hypertension (19, 24, 35). Gene-based analysis is a method that can evaluate the association between a trait and a candidate gene, and is performed with *R* software (version 3.0.1; http://www.r-project.org).

For the analyses of the cross-sectional cohort study, χ^2^-test, Student's *t*-test and Mann–Whitney test were used to compare the difference between groups as appropriate. One-way ANOVA was used to test the linearity across different hypertension grades and subtypes. We performed linear and logistic regression analyses to examine the associations of serum uromodulin levels with BP levels and the risk of hypertension. Multivariable models were adjusted for traditional cardiovascular risk factors and potential confounders. All statistical analyses were performed using SPSS 16.0 for windows (SPSS, Inc., Chicago, IL). *P* < 0.05 was considered statistically significant.

## Results

### Characteristics of the Study Participants at Baseline and During Follow-Ups in the Longitudinal Cohort Study

At baseline, the age of the participants was 48.6 years, BMI was 22.2 kg/m^2^, and the mean SBP, DBP, and MAP were 115.2, 71.3, and 86.0 mmHg, respectively. 51 (9.9%) subjects were diagnosed with hypertension at baseline. After 8 years of follow-up, the mean SBP, DBP, and MAP increased by 14.4, 6.6, and 9.1 mmHg, respectively, and 103 (28.9%) subjects developed hypertension (Table 1).

**Table 1:** Characteristics of the study participants at baseline and during follow-ups in the longitudinal cohort study.

**Characteristics**	**Baseline**	**Follow-up**	**Follow-up**
	**in 2004**	**in 2009**	**in 2012**
Gender (M/F)	267/247	208/204	185/171
Age (years)	48.6 ± 19.8	53.3 ± 14.2	56.6 ± 19.0
Body mass index (kg/m^2^)	22.2 ± 3.1	22.4 ± 3.3	23.6 ± 3.5
SBP (mmHg)	115.2 ± 17.6	120.0 ± 17.3	129.6 ± 18.7
DBP (mmHg)	71.3 ± 10.0	75.8 ± 10.4	77.9 ± 10.9
MAP (mmHg)	86.0 ± 11.5	90.5 ± 11.7	95.1 ± 11.9
Hypertension at baseline (*n*, %)	51 (9.9)	–	–
Hypertension incidence (*n*, %)^*^	–	77 (18.9)	103 (28.9)

*Data are expressed as mean ± SD or n, %. SBP, systolic blood pressure; DBP, diastolic blood pressure; MAP, mean arterial pressure*.

* *Participants with hypertension at baseline were excluded*.

### The Association of *UMOD* With the Longitudinal BP Changes and the Incidence of Hypertension

Information on genotyped SNPs, including the genomic location, minor allele frequency, Hardy-Weinberg test and potential function prediction, are shown in Supplementary Table 1. No SNP deviated significantly from Hardy-Weinberg equilibrium.

The associations of each SNP in *UMOD* gene with the 5-year (2004–2009) and 8-year (2004–2012) BP changes are presented (Table 2). *UMOD* SNP rs12917707 and rs12708631 were significantly associated with the longitudinal changes in SBP, DBP, MAP and PP at both follow-ups. SNP rs11859916 was significantly associated with the 8-year change in PP. In addition, SNP rs12708631 was significantly associated with the incidence of hypertension (*OR* = 1.344, *P* = 0.035) over 8 years (Table 3). Gene-based analyses further showed that *UMOD* gene was significantly associated with the longitudinal SBP changes (*P*_TPM_ = 0.004), MAP changes (*P*_TPM_ = 0.035), PP changes (*P*_TPM_ = 0.019) and hypertension incidence (*P*_TPM_ = 0.044) over the 8-year follow-up after adjustment for multiple testing.

**Table 2:** Association of *UMOD* SNPs with longitudinal blood pressure changes changes blood pressure from baseline to the follow-ups.

**SNP**	**BP(2004–2009)**				**BP(2004–2012)**			
	**SBP change**	**DBP change**	**MAP change**	**PP change**	**SBP change**	**DBP change**	**MAP change**	**PP change**
rs4632135	0.487	0.885	0.665	0.394	1.000	0.649	0.788	0.722
rs4383153	0.487	0.885	0.665	0.394	1.000	0.649	0.788	0.722
rs11859916	0.110	0.349	0.178	0.140	0.160	0.430	0.240	**0.041^a^**
rs7198000	0.162	0.421	0.242	0.195	0.352	0.533	0.402	0.452
rs7193058	0.186	0.431	0.262	0.227	0.094	0.274	0.136	0.171
rs77875418	0.577	0.427	0.459	0.889	0.217	0.300	0.218	0.407
rs79245268	0.538	0.412	0.433	0.841	0.120	0.224	0.133	0.264
rs4293393	0.562	0.430	0.454	0.861	0.112	0.134	0.092	0.344
rs6497476	0.538	0.412	0.433	0.841	0.120	0.224	0.133	0.264
rs4997081	0.745	0.730	0.716	0.860	0.378	0.341	0.314	0.667
rs13333226	0.516	0.388	0.408	0.833	0.087	0.100	0.066	0.320
rs12708631	**0.012*^b^***	**0.006** *^***b***^*	**0.004*^b^***	0.166	**0.030*^a^***	**0.020*^b^***	**0.020**	0.236
rs12917707	**0.009**	0.600	0.107	**0.001**	**0.023*^b^***	0.726	0.403	**0.002**

*For associations that were not significant under any model, P values for an additive model are listed. All genetic models are based on the minor allele of each SNP. BP, blood pressure; SBP, systolic blood pressure; DBP, diastolic blood pressure. MAP, mean arterial pressure; PP, pulse pressure; OR, odds ratio; SNP, single nucleotide polymorphism*.

a *Dominant model*.

b *Recessive model*.

*Statistically values are presented in bold*.

**Table 3:** Association of *UMOD* individual SNPs with hypertension incidence.

**SNP**	**Incident hypertension**		**Incident hypertension**	
	**(2004–2009)**		**(2004–2012)**	
	***OR***	***P* value**	***OR***	***P* value**
rs4632135	0.856	0.634	1.215	0.473
rs4383153	0.856	0.634	1.215	0.473
rs11859916	1.281	0.272	1.395	0.101
rs7198000	1.197	0.430	1.268	0.243
rs7193058	1.232	0.441	1.459	0.114
rs77875418	1.147	0.733	1.294	0.472
rs79245268	1.094	0.822	1.358	0.387
rs4293393	0.913	0.818	1.364	0.355
rs6497476	1.094	0.822	1.358	0.387
rs4997081	1.393	0.287	0.828	0.470
rs13333226	0.934	0.864	1.420	0.301
rs12708631	1.066	0.731	**1.344**	**0.035**
rs12917707	0.903	0.647	0.755	0.156

*For associations that were not significant under any model, P values for an additive model are listed. All genetic models are based on the minor allele of each SNP. OR, odds ratio; SNP, single nucleotide polymorphism. Statistically values are presented in bold*.

### Characteristics of the Study Population in the Cross-Sectional Study

Participants with hypertension were older, and are more likely to be men and to have higher BMI. Smoking, alcohol consumption, diabetes, and family history of hypertension were more common in the hypertension vs. normotension group. Compared with the normotension group, the following biochemical markers were higher in the hypertension group: SUA; blood glucose; ALT; AST; total cholesterol; triglycerides; LDL; urinary albumin/creatinine; and serum creatinine. In contrast, eGFR and HDL were lower in the hypertensive group (Table 4).

**Table 4:** Characteristics of participants categorized by BP status in the cross-sectional cohort study (*n* = 2,210).

**Characteristics**	**All**	**Normotensive**	**Hypertensive**	***P*-value**
No. of subjects	2,210	1,764	446	–
Age (years)	42.7 (40.0–45.0)	42.6 (40.0–45.0)	43.3 (41.0–45.0)	0.002
Gender (M/F)	1,197/1,013	874/890	323/123	<0.001
BMI (kg/m^2^)	24.1(21.9–26.0)	23.3 (21.5–25.3)	25.7 (23.7–27.7)	<0.001
SBP (mmHg)	121.0 (112.0–130.8)	117.7 (110.0–125.3)	142.3 (133.9–153.0)	<0.001
DBP (mmHg)	75.7 (69.0–83.7)	73.7 (67.3–79.3)	90.7 (85.7–96.7)	<0.001
Heart rate (beats/min)	73.0 (66.0–80.0)	72.5 (66.0–79.0)	75.0 (69.0–83.0)	<0.001
Alcohol consumption (*n*, %)	629 (28.5)	454 (25.7)	175 (39.2)	<0.001
Current smoking (*n*, %)	929 (42.0)	674 (38.2)	255 (57.2)	<0.001
Diabetes mellitus (*n*, %)	95 (4.3)	63 (3.6)	32 (7.2)	0.001
FH of hypertension (*n*, %)	1146 (51.9)	851 (48.2)	295 (66.1)	<0.001
Education level (*n*, %)				0.762
Primary school or less	119 (5.4)	97 (5.5)	22 (4.9)	
Middle school	1406 (63.7)	1120 (63.6)	286 (64.1)	
High school	482(21.8)	380 (21.6)	102 (22.9)	
College or more	201 (9.1)	165 (9.4)	36 (8.1)	
Marital status (*n*, %)				0.073
Married	2099 (95.0)	1679 (95.2)	420 (94.2)	
Divorced	81 (3.7)	66 (3.7)	15 (3.4)	
Unmarried or Other	30 (1.4)	19 (1.1)	11 (2.5)	
Level of physical activity (*n*, %)				0.669
Almost no	903 (40.9)	720(41.8)	183 (41.0)	
Light	1166 (52.8)	930 (52.7)	236 (52.9)	
Moderate	90 (4.1)	70 (4.0)	20 (4.5)	
Heavy	51 (2.3)	44 (2.5)	7 (1.6)	
Serum uric acid (μmol/L)	277.9 (225.2–333.1)	269.3 (218.6–319.5)	322.2 (264.9–372.1)	<0.001
Fasting glucose (mmol/L)	4.56 (4.27–4.90)	4.53 (4.24–4.85)	4.73 (4.38–5.11)	<0.001
ALT (U/L)	18.0 (13.0–27.0)	18.0 (13.0–25.0)	23.0 (16.0–32.0)	<0.001
AST (U/L)	16.0 (13.0–20.0)	16.0 (13.0–20.0)	18.0 (14.0–23.0)	<0.001
Total cholesterol (mmol/L)	4.51 (4.05–5.00)	4.49 (4.02–4.97)	4.61 (4.19–5.15)	<0.001
Triglycerides (mmol/L)	1.32 (0.95–1.93)	1.25 (0.91–1.80)	1.66(1.19–2.35)	<0.001
LDL (mmol/L)	2.49 (2.13–2.90)	2.48(2.11–2.86)	2.54 (2.23–2.99)	0.001
HDL (mmol/L)	1.15 (0.99–1.34)	1.17 (1.00–1.36)	1.08 (0.96–1.24)	<0.001
Serum creatinine (μmol/L)	75.874.5 (66.6–86.2)	74.5 (65.7–84.9)	81.7 (71.4–89.5)	<0.001
eGFR (mL/min/1.73 m^2^)	85.4 (72.6–100.2)	87.4 (73.9–101.6)	78.1 (69.4–91.8)	<0.001
Urine albuinin/creatinine (mg/g)	8.70(5.63–15.21)	8.04 (5.31–13.14)	13.72 (7.65–31.02)	<0.001

*BP, blood pressure; BMI, body mass index; SBP, systolic blood pressure; DBP, diastolic blood pressure; FH, Family history; ALT, alanine aminotransferase; AST, aspartate transaminase; LDL, low-density lipoprotein; HDL, high-density lipoprotein; eGFR, estimated glomerular filtration rate; Non-normally distributed variables are expressed as the median (interquartile range). All other values are expressed as mean ± SD or n, %*.

### Associations of Serum Uromodulin With BP Levels and the Risk of Hypertension

**Figure 1:**
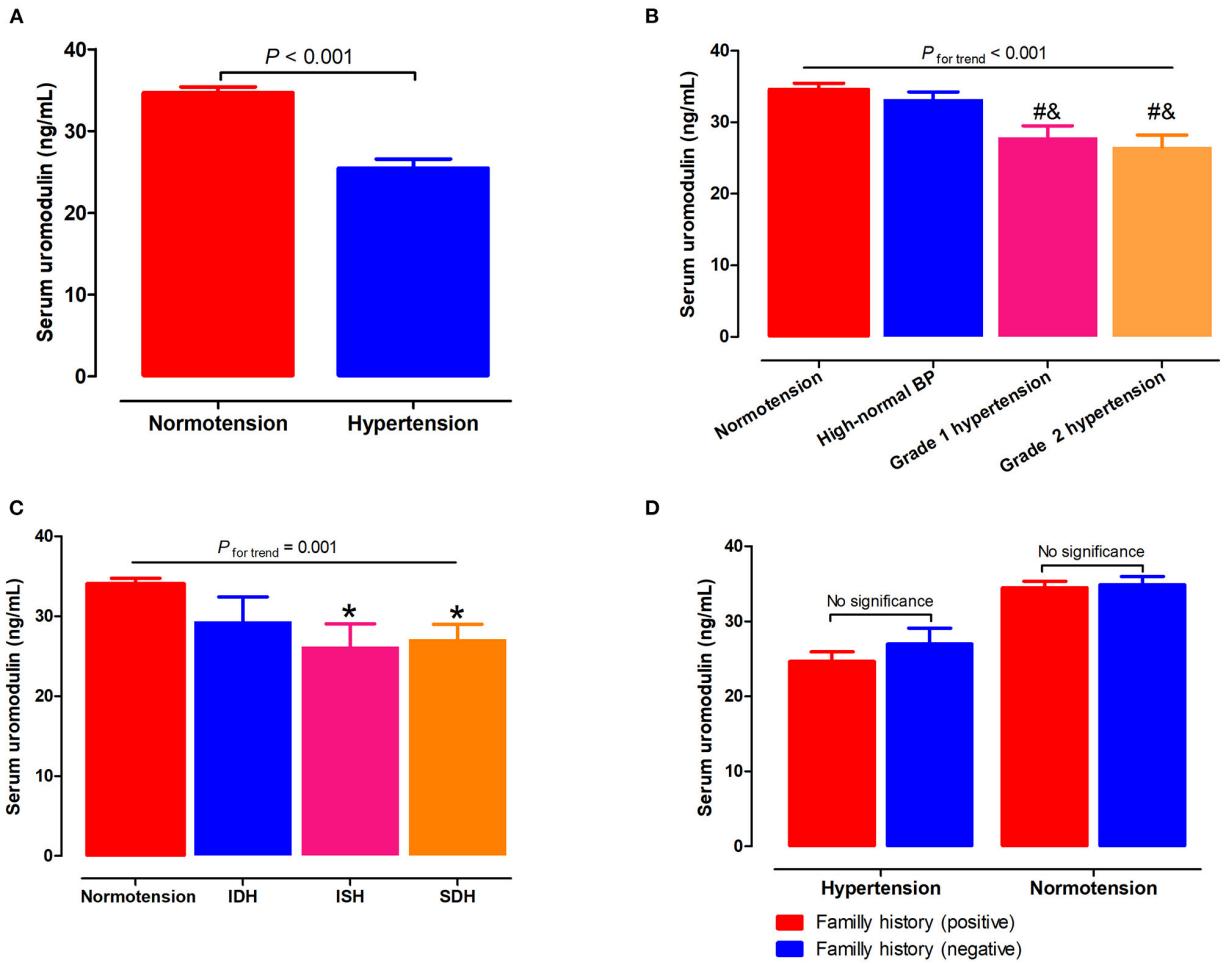
Associations of serum uromodulin with BP levels and hypertension. **(A)** Serum uromodulin levels in subjects with normotension and hypertension. **(B)** Serum uromodulin levels in subjects with different grades of BP, including normotension, high-normal, grade 1 hypertension, and grade 2 hypertension. ^#^*P* < 0.05 *vs*. normotension, ^&^*P* < 0.05 *vs*. high-normal BP. **(C)** Serum uromodulin levels in subjects with normotension and different hypertensive subtypes, including ISH, IDH, SDH, and those with controlled and uncontrolled BP in the absence of antihypertensive treatment. *P < 0.05 *vs*. normotension. **(D)** Serum uromodulin levels in hypertensive or normotensive subjects with a family history of hypertension and those without. BP, blood pressure; ISH, isolated systolic hypertension (SBP ≥ 140 mmHg and DBP <90 mmHg); IDH, isolated diastolic hypertension (SBP <140 mmHg and DBP ≥ 90 mmHg); SDH, systolic diastolic hypertension (SBP ≥ 140 mmHg and DBP ≥ 90 mmHg).

Serum uromodulin levels were significantly lower in hypertensive subjects than in the normotensive subjects (25.5 ± 1.1 *vs*. 34.7 ± 0.7ng/mL, *P* < 0.001; Figure 1A). Next, we assessed serum uromodulin levels in different grades of hypertension, which was presented in Figure 1B. Serum uromodulin levels decreased gradually as BP levels increased (34.6, 33.2, 27.8, and 25.0 ng/mL for subjects with normotension, high-normal, grade 1 hypertension, and grade 2 hypertension, respectively, *P*_fortrend_ < 0.001). We further examined serum uromodulin in different groups with normotensive and hypertensive subtypes. Participants with ISH and SDH have lower serum uromodulin levels than normotensive subjects. Serum uromodulin was 34.1, 29.3, 26.2, and 27.1 ng/mL for subjects with normotension, IDH, ISH, and SDH, respectively (*P*_fortrend_ = 0.001, Figure 1C). In addition, we further divided all hypertensive and normotensive subjects into two groups according to the family history. No significant difference in serum uromodulin was found between any groups (Figure 1D).

**Figure 2:**
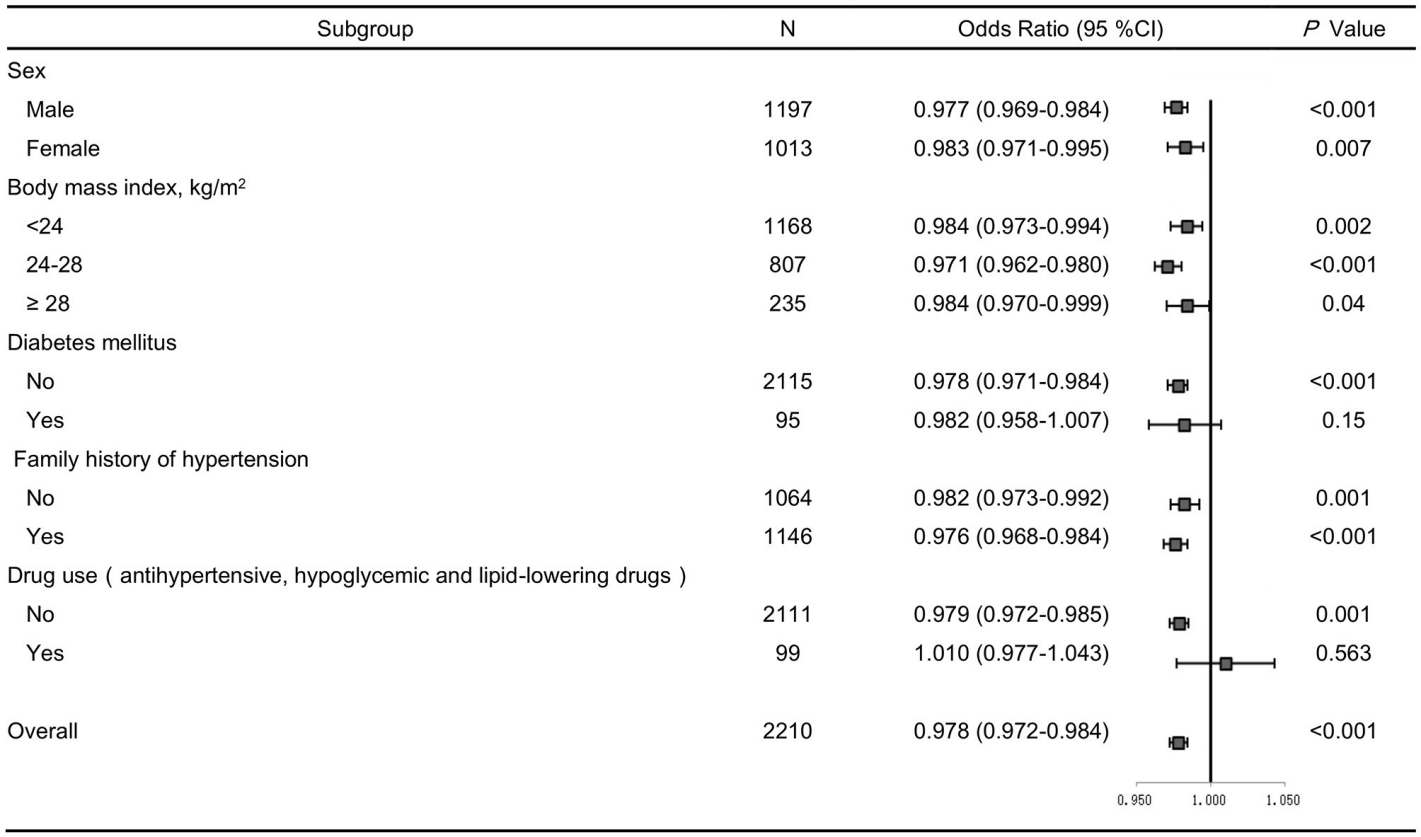
Forest plots of odds ratios (ORs) for serum uromodulin and risk of hypertension after adjustment. The adjustment model includes age, smoke, BMI, serum creatinine, total cholesterol, triglycerides, fasting glucose in subjects stratified by sex, BMI, diabetes, family history of hypertension, and drug use. Values are the OR (95% confidence interval [95% CI]).

SBP levels positively correlated with age, BMI, glucose, serum creatinine and total cholesterol, but inversely correlated with gender and serum uromodulin (β = −0.075, *P* = 0.001). In addition, DBP levels were also positively correlated with age, BMI, glucose, serum creatinine and total cholesterol, but negatively correlated with gender and serum uromodulin (β = −0.084, *P* < 0.001, Table 5). Furthermore, serum uromodulin was significantly associated with a lower risk of hypertension after adjusting for multiple confounders [0.978 (0.972–0.984), *P* < 0.001; Figure 2].

**Table 5:** Relationships between various characteristics and BP levels (*n* = 2,210).

**Characteristics**	**SBP**		**DBP**	
	**β**	***P* value**	**β**	***P* value**
Serum uromodulin (ng/mL)	−0.075	<0.001	−0.084	<0.001
Age (years)	0.113	<0.001	0.064	0.001
Gender (M/F)	−0.135	<0.001	−0.200	<0.001
Current smoking (*n*, %)	0.018	0.521	−0.020	0.464
BMI (kg/m^2^)	0.306	<0.001	0.283	<0.001
Fasting glucose (mmol/L)	0.065	0.001	0.061	0.002
Serum creatinine (μmol/L)	0.085	<0.001	0.091	<0.001
Total cholesterol (mmol/L)	0.040	0.040	0.046	0.021
Triglycerides (mmol/L)	0.023	0.273	0.035	0.093

*SBP, systolic blood pressure; DBP, diastolic blood pressure; BMI, body mass index*.

We further performed several sensitivity analyses to test the robustness of the analysis. After excluding participants with diabetes, or those taking antihypertensive, hypoglycemic and lipid-lowering drugs, similar results were obtained (Figure 2). In addition, we stratified all participants by sex, BMI, family history of hypertension, and the results remained the same (Figure 2).

## Discussion

Previous animal studies showed that uromodulin may be involved in BP regulation and the development of hypertension. Graham et al. (36) found that *UMOD* knockout (*UMOD*^−/−^) mice had significantly lower SBP than wild-type mice and were resistant to salt-induced BP changes. In the *UMOD* knockout mice, the pressure–natriuresis curve shifted to the left. By contrast, Trudu et al. (37) showed that *UMOD* overexpression increased BP in a dose-dependent manner due to the increased *UMOD* expression and excretion. These studies suggest that uromodulin may affect the development of hypertension by modifying sodium transport in the TAL. A previous clinical study also has shown a link between *UMOD* gene and hypertension. A large genome-wide association studies (GWAS) from the European cohorts showed that the minor G allele of rs13333226 had a lower risk of hypertension (17). By contrast, Algharably et al. (38) found that no significant associations between rs12917707 and mean 24 h SBP or DBP or any other BP phenotype were detected a cohort of 1,218 white individuals. Our long-term follow-up study showed for the first time that novel *UMOD* markers rs12708631 and rs12917707 were associated with longitudinal BP changes. In addition, this is the first study to explore the association between *UMOD* and the incidence of hypertension over time. *UMOD* variant rs12708631 was significantly associated with the incidence of hypertension during the 8-year follow-up. In the gene-based analyses, *UMOD* was aggregately associated with the incidence of hypertension. The different study populations, sample sizes, and racial differences among these various studies may be the causes of the discrepant results. The associations of *UMOD* gene variants with hypertension susceptibility may be linked to the role of uromodulin in regulating sodium-potassium-chloride cotransporter NKCC2 in the TAL and suggest that uromodulin is a potential therapeutic target for hypertension (39).

Several studies have shown that urinary uromodulin was independently associated with a rapid decline in renal function and the incidence of end-stage renal disease (ESRD) (40–42). While most studies in the past have focused on urinary uromodulin, few studies have looked at uromodulin in blood. Recently, Steubl et al. and Bostom et al. reported the associations of blood uromodulin levels with renal function and recommended its use as a potential kidney biomarker (14, 43, 44). In our study, subjects with hypertension had significantly lower serum uromodulin levels than those without hypertension. In contrast to the previous studies that used only the dichotomous definitions of hypertension, our study reports grade-specific hypertension pursuant to the 2020 ISH guideline classification. To our knowledge, ours is the first study to apply such definitions. We found that serum uromodulin showed a linear decrease from normotension to grade 2 hypertension, suggesting that circulating uromodulin may be a novel marker for identifying stages of hypertension. In addition, our study is the first to explore serum uromodulin levels in different hypertension subtypes. ISH is usually characterized as an aging phenomenon. Unlike DBP, SBP increases with age as arterial stiffness increases and arterial compliance decreases (45). SBP has been shown to be a more reliable predictor of adverse cardiovascular events than DBP (46, 47). In the present study, serum uromodulin in the ISH and SDH groups was significantly lower than the normotension group, but was similar to the IDH group. These data indicate serum uromodulin may be an independent marker of hypertension that identifies its subtypes and grades.

The current study has several strengths. First, we performed stringent quality control procedures in genotyping and data collection. At baseline and each follow-up survey, we used the mean values of 9 separate BP measurements for the final analysis, thereby reducing measurement errors. Furthermore, our study comprehensively examined the associations of serum uromodulin levels with hypertension and its subtypes and grades. However, several limitations should be acknowledged. The novel findings in our study need to be replicated in other cohorts of different genetic backgrounds. Additionally, due to the limited number of genotyped SNPs in the *UMOD* gene in this study, less frequent genetic variants may have been omitted.

In conclusion, based on both single-marker and gene-based analyses, we report for the first time that *UMOD* gene was associated with longitudinal BP phenotypes and hypertension incidence. Furthermore, our study also showed that serum uromodulin level was significantly associated with hypertension and its subtypes and grades. These findings suggest that serum uromodulin may serve as a biomarker marker for hypertension. In addition, this work contributes to the accumulating evidence that genomic differences regulate BP and the development of hypertension.

## Data Availability Statement

The raw data supporting the conclusions of this article will be made available by the authors, without undue reservation.

## Ethics Statement

The studies involving human participants were reviewed and approved by the Ethics Committee of the First Affiliated Hospital of Xi'an Jiaotong University. The patients/participants provided their written informed consent to participate in this study.

## Author Contributions

YW and J-JM conceived and designed the experiments. J-JM was responsible for subject recruitment. YW, X-YZ, TZ, CChu, CChen, DW, Y-YL, QM, K-KW, Z-JN, R-CY, YY, H-WZ, HJ, W-HG, HL, C-HL, KG, and JZ performed the experiments. T-LY, SY, F-YC, YW, and M-FD analyzed the data. YW, M-FD, and SY drafted the paper. FW, RS, and J-JM edited and revised manuscript. All authors have read, critically revised, and approved the final manuscript.

## Funding

This work was supported by the National Natural Science Foundation of China Nos. 81600327 (YW) and 81870319, 82070437 (J-JM), Natural Science Basic Research Program of Shaanxi Province (2021JM-257, 2021JM-588), Grants from China Postdoctoral Science Foundation funded project (Nos. 2018T111075 and 2018M631177), Institutional Foundation of the First Affiliated Hospital of Xi'an Jiaotong University No. 2019QN-06, Grants from the Major Chronic Non-communicable Disease Prevention and Control Research Key Project of the Ministry of Science and Technology of China (2017YFC1307604), and the Clinical Research Award of the First Affiliated Hospital of Xi'an Jiaotong University of China No. XJTU1AF-CRF-2019-004.

## Conflict of Interest

The authors declare that the research was conducted in the absence of any commercial or financial relationships that could be construed as a potential conflict of interest.

## Publisher's Note

All claims expressed in this article are solely those of the authors and do not necessarily represent those of their affiliated organizations, or those of the publisher, the editors and the reviewers. Any product that may be evaluated in this article, or claim that may be made by its manufacturer, is not guaranteed or endorsed by the publisher.
