# Supplementary material for: Associations of Serum Uromodulin and Its Genetic Variants With Blood Pressure and Hypertension in Chinese Adults
Source: Front Cardiovasc Med. 2021 Nov 17;8:710023. doi: 10.3389/fcvm.2021.710023 (PMC8635522; doi:10.3389/fcvm.2021.710023)
Supplement: Supplementary file 1 [file Data_Sheet_1.docx]

Supplementary Material

# Supplementary figures and Tables

## Supplementary Figures

**
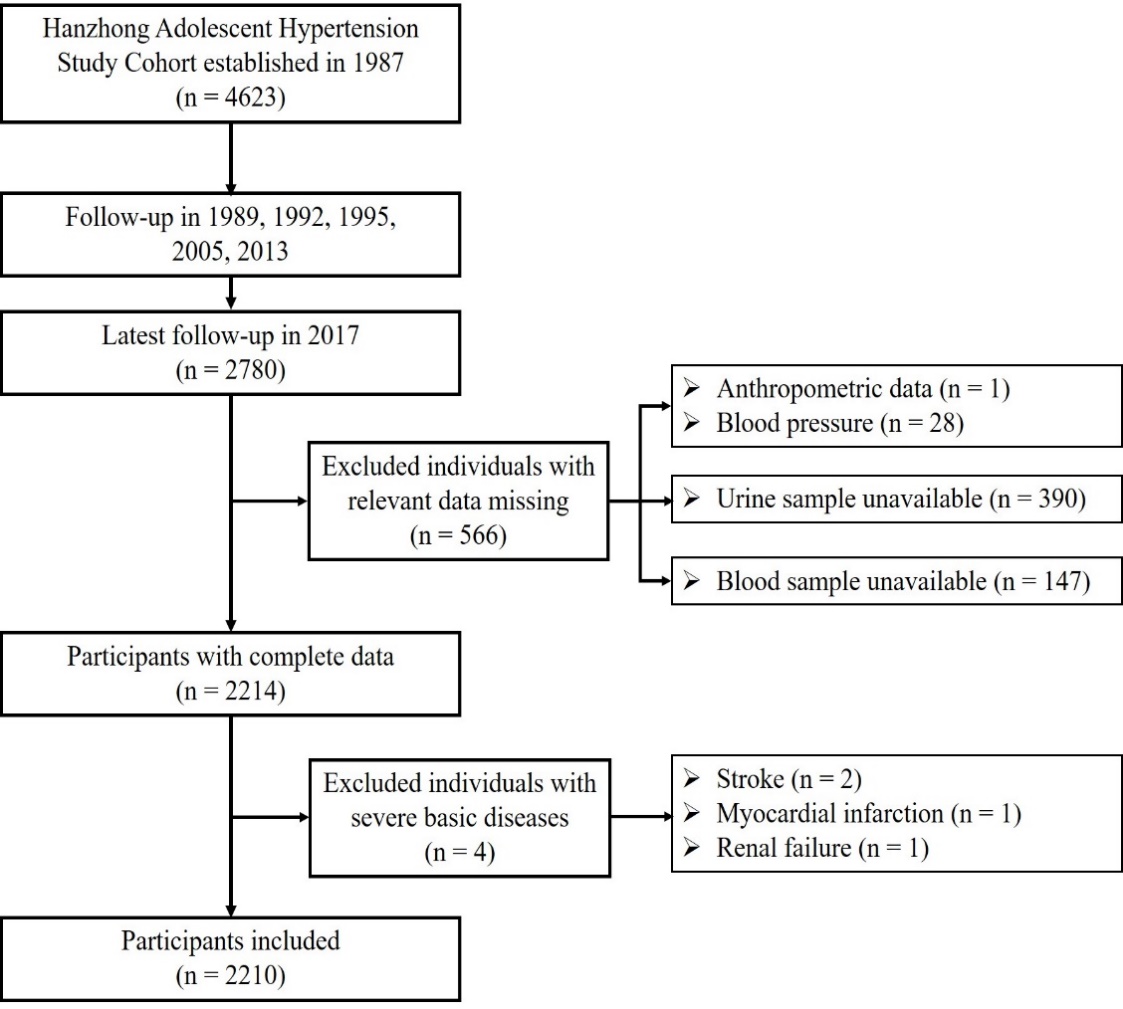
**

**Figure S1.** Flow diagram for recruitment of participants in cross-sectional study.

## Supplementary Tables

**Table S1.** Information on genotyped SNPs of *UMOD*

| **SNP** | **Position** | **Region** | **Alleles *^a^*** | | **MAF** | ***P*-value*^b^*^†^** | **Potential function prediction** |
| --- | --- | --- | --- | --- | --- | --- | --- |
| rs4632135 | 20337884 | intronic | | T/C | 0.105 | 1 | DHS |
| rs4383153 | 20338622 | intronic | | G/A | 0.105 | 1 | TFBS |
| rs11859916 | 20351231 | intronic | | G/A | 0.203 | 0.815 | DHS |
| rs7198000 | 20351937 | intronic | | G/A | 0.193 | 0.808 | DHS |
| rs7193058 | 20360101 | exonic | | G/A | 0.248 | 0.384 | TFBS; DHS |
| rs77875418 | 20360359 | exonic | | G/A | 0.051 | 1 | TFBS; DHS |
| rs79245268 | 20362115 | exonic | | C/T | 0.051 | 1 | DHS |
| rs4293393 | 20364588 | intronic | | A/G | 0.057 | 1 | DHS |
| rs6497476 | 20364781 | intronic | | T/C | 0.051 | 1 | TFBS |
| rs4997081 | 20365234 | intronic | | C/G | 0.382 | 0.079 | - |
| rs13333226 | 20365654 | intronic | | A/G | 0.057 | 1 | - |
| rs12708631 | 20365697 | intronic | | T/A | 0.397 | 0.748 | - |
| rs12917707 | 20381234 | intronic | | G/A | 0.1422 | 0.483 | - |

SNP, single nucleotide polymorphism; MAF, minor allele frequency; DHS, DNase I hypersensitive sites; TFBS, transcription factor binding site. ^†^parents only (parental generation). *^b^P* values of Hardy-Weinberg equilibrium test. *^a^*Alleles are presented as major: minor allele.
